# Feasibility of neuromuscular electrical stimulation in critically ill patients

**DOI:** 10.1186/cc12473

**Published:** 2013-03-19

**Authors:** J Segers, G Hermans, F Bruyninckx, G Meyfroidt, D Langer, R Gosselink

**Affiliations:** 1 UZ/KU Leuven, Belgium

## Introduction

Survivors of critical illness often have a prolonged stay on the ICU. These patients may suffer from ICU-acquired weakness. It has been shown that reduction in muscle mass and muscle strength occurs early after admission to the ICU. However, in the very early stage on the ICU, patients are often sedated and not able to participate in any active mobilizations. Therefore the use of neuromuscular electrical stimulation (NMES) is becoming a treatment of interest in the ICU. The aim was to study the feasibility and safety of NMES in a surgical and medical ICU of a large, tertiary referral university hospital.

## Methods

Fifty patients with an expected prolonged stay on the ICU of 5 more days (judged on day 3) with no trauma or neurological disease were included. They then received daily a NMES session (DUO 500; Gymna, Belgium) for 25 minutes on the quadriceps bilaterally during their entire stay on the ICU. The main outcome was the ability to produce a contraction of the quadriceps through NMES. The muscle contraction was quantified on a 5-point scale: 1 (no contraction palpable and visible) up to 5 (contraction very well palpable and visible). Patients were classified as responders when an adequate muscle bulk was obtained in ≥75% of the sessions. The potential factors associated with the feasibility were: gender, age, body mass index (BMI), diagnosis of sepsis, Barthel index prior to admission to the hospital, APACHE II score, Glasgow Coma Scale (GCS), five questions for adequacy, stimulus intensity and leg edema. A multiple regression analysis was performed to identify the factors determining whether or not a contraction could be expected in a patient. Safety of NMES was assessed through heart rate, blood pressure, oxygen saturation and respiratory rate.

## Results

In 48% of the patients we were able to achieve adequate muscle contractions in more than 75% of the sessions. GCS (*P *= 0.047), edema (*P *= 0.001) and sepsis (*P *= 0.010) were significantly different between responders and nonresponders. Responders had a lower mean GCS (7 ± 3 vs. 9 ± 3), lower amount of edema and were less likely to have had sepsis. In a multiple regression analysis, sepsis, edema, BMI and age explained 51% of the variance. As for safety, none of the parameters changed significantly.

## Conclusion

In patients with a better neurological condition, sepsis and/or leg edema it was more difficult to obtain an adequate quadriceps contraction with NMES. NMES is safe to apply on the ICU.

